# Endoscopic *versus* surgical treatment of ampullary adenomas: a systematic review and meta-analysis

**DOI:** 10.6061/clinics/2016(01)06

**Published:** 2016-01

**Authors:** Ernesto Quaresma Mendonça, Wanderley Marques Bernardo, Eduardo Guimarães Hourneaux de Moura, Dalton Marques Chaves, André Kondo, Leonardo Zorrón Cheng Tao Pu, Felipe Iankelevich Baracat

**Affiliations:** I Faculdade de Medicina da Universidade de São Paulo, Gastroenterologia, Unidade de Endoscopia Gastrointestinal, São Paulo/SP, Brazil.; II CEDEM - Centro de Desenvolvimento de Educação Médica, São Paulo/, SP, Brazil; III Brazilian Medical Association - Guidelines Developing, São Paulo/, SP, Brazil

**Keywords:** Ampullary Adenoma, Ampulla of Vater, Duodenal Neoplasms, Endoscopy, Pancreaticoduodenectomy, Surgery

## Abstract

The aim of this study is to address the outcomes of endoscopic resection compared with surgery in the treatment of ampullary adenomas.

A systematic review and meta-analysis were performed according to Preferred Reporting Items for Systematic Reviews and Meta-Analyses (PRISMA) recommendations. For this purpose, the Medline, Embase, Cochrane, Literatura Latino-Americana e do Caribe em Ciências da Saúde (LILACS), Scopus and Cumulative Index to Nursing and Allied Health Literature (CINAHL) databases were scanned. Studies included patients with ampullary adenomas and data considering endoscopic treatment compared with surgery. The entire analysis was based on a fixed-effects model.

Five retrospective cohort studies were selected (466 patients). All five studies (466 patients) had complete primary resection data available and showed a difference that favored surgical treatment (risk difference [RD] = -0.24, 95% confidence interval [CI] = -0.44 to -0.04). Primary success data were identified in all five studies as well. Analysis showed that the surgical approach outperformed endoscopic treatment for this outcome (RD = -0.37, 95% CI = -0.50 to -0.24). Recurrence data were found in all studies (466 patients), with a benefit indicated for surgical treatment (RD = 0.10, 95% CI = -0.01 to 0.19). Three studies (252 patients) presented complication data, but analysis showed no difference between the approaches for this parameter (RD = -0.15, 95% CI = -0.53 to 0.23).

Considering complete primary resection, primary success and recurrence outcomes, the surgical approach achieves significantly better results. Regarding complication data, this systematic review concludes that rates are not significantly different.

## INTRODUCTION

Neoplasms of the duodenal papilla are not a common condition. In studies with autopsy series, there is a reported prevalence of 0.04% to 0.12% 1,2. As with colon cancer, there appears to be an adenoma-to-carcinoma progression sequence in ampullary adenomas and the rate of development of carcinoma from adenoma has been shown to be 30% 3. However, the time frame for malignant transformation is not well established 4. Due to this malignant potential, complete removal of an adenoma is essential for curative therapy 5.

Pancreaticoduodenectomy (PD) or pylorus-preserving PD (PpPD) has traditionally been performed to treat tumors of the ampulla of Vater. However, given the high morbidity and mortality associated with radical surgery and the adequacy of local resection for ampullary adenomas, endoscopic papillectomy (EP) has been established as a safe, effective and reliable treatment modality for benign tumors of the ampulla, serving as an alternative to surgery 6. The issue today is not whether ampullary adenomas *can* be endoscopically resected, but rather, which cases *should* be resected. Therefore, accurate preoperative evaluation is necessary, although this is not always feasible.

The false-negative rate of endoscopic biopsy for cancer has been reported to be high, ranging from 11.7% to 60% 7 and the coexistence of carcinoma within adenoma cannot be excluded by pre-procedural biopsy 8,9. Considering this, preoperative evaluation must include not only biopsy but also the size of the tumor, echographic study of the region and endoscopic findings.

It is agreed that the indication for EP is an adenoma of the papilla of Vater that is confined to the ampullary region 10. Therefore, endoscopic snare papillectomy can be performed on a tumor that has not invaded the muscularis propria of the duodenum; it is not indicated when the tumor extends into the biliary common duct for more than 10 mm. Lesions that fulfill this criterion can also be treated with surgery, especially when dysplasia is present in the pre-procedure biopsy, when ductal dilation without ductal invasion in the endoscopic ultrasound (EUS) evaluation, or when the tumor is large in size (more than 4 cm), although the clinical condition of the patient must be considered.

The management strategies for ampullary adenoma are expanding; nevertheless, the criteria for its surgical treatment have not yet been established. Considering the scenario of difficult preoperative evaluation and the lack of a consensus and guidelines, it is very hard to find studies that compare the results of surgical and endoscopic resection in patients with ampullary adenomas, so this type of comparison has not yet been reported in systematic reviews.

To address the outcomes of endoscopic resection compared with surgery in the treatment of ampullary adenoma, we developed and performed this systematic review.

## MATERIALS AND METHODS

This systematic review of the literature was conducted in accordance with the Preferred Reporting Items for Systematic Reviews and Meta-analyses (PRISMA) 11 recommendations and was registered in the PROSPERO 12 international database (www.crd.york.ac.uk/prospero/) under number CRD42014015311.

### Study identification and selection

The eligibility criteria for the studies included in this review are shown in Table 1. Studies were identified by searching electronic databases and scanning the reference lists of articles. No limits were applied for language. The search was applied in Medline (considering all years), and in Embase (considering all years), a shortened strategy was needed. The Cochrane and Literatura Latino-Americana e do Caribe em Ciências da Saúde (LILACS) (via BVS) and the Scopus and Cumulative Index to Nursing and Allied Health Literature (CINAHL) (via EBSCO) databases were also reviewed. The last manual search was run on October 1^st^, 2014. For Medline, automated updates based on the search strategy were evaluated for new studies monthly until September 2015. Eligibility assessment and the selection of screened records were performed independently in an unblinded standardized manner by two reviewers.

The following search strategy was used for the Medline database: ‘(Adenoma OR Ampulla of Vater OR Duodenal Neoplasms OR Common Bile Duct Neoplasms) AND (Endoscopy OR Duodenoscopy OR Endoscopy, Gastrointestinal) AND (Pancreaticoduodenectomy OR Surgical Procedures, Elective OR Laparotomy OR Surgery) AND ((clinical[Title/Abstract] AND trial[Title/Abstract]) OR clinical trials as topic[MeSH Terms] OR clinical trial[Publication Type] OR random*[Title/Abstract] OR random allocation[MeSH Terms] OR therapeutic use[MeSH Subheading]) OR Comparative study OR Comparative studies OR Random*) NOT (Colonoscopy OR Colonic Neoplasms OR Colorectal Neoplasms OR Rectal Neoplasms OR Anal Canal OR pituitary surgery OR Pituitary Neoplasms OR Lung Neoplasms OR Pleural Neoplasms OR Mesothelioma OR Skull Base OR Neurosurgical Procedures OR Parathyroidectomy OR Neck OR Hyperparathyroidism OR Adrenalectomy OR Adrenal Gland Diseases)'. As part of the process, the Medline search strategy was peer reviewed.

The Embase search was as follows: ‘(Adenoma OR Ampulla of Vater OR Duodenal Neoplasms OR Common Bile Duct Neoplasms) AND (Endoscopy OR Duodenoscopy OR Endoscopy, Gastrointestinal) AND (Pancreaticoduodenectomy OR Surgical Procedures, Elective OR Laparotomy OR Surgery) NOT (Colonoscopy OR Colonic Neoplasms OR Colorectal Neoplasms OR Rectal Neoplasms OR Anal Canal OR pituitary surgery OR Pituitary Neoplasms OR Lung Neoplasms OR Pleural Neoplasms OR Mesothelioma OR Skull Base OR Neurosurgical Procedures OR Parathyroidectomy OR Neck OR Hyperparathyroidism OR Adrenalectomy OR Adrenal Gland Diseases)'.

For the Cochrane, LILACS, Scopus and CINAHL databases, the search was ‘Duodenal Neoplasms AND Endoscopy AND Surgical Procedures'.

### Data collection process

The method of data extraction from each included study consisted of filling out an information sheet after the paper was read. A Scottish Intercollegiate Guidelines Network (SIGN) (13)-based checklist was gathered from the internet site www.sign.ac.uk. Relevant data were then extracted from each included study using a standardized extraction form. One review author extracted the data mentioned below from the included studies and a second author checked the extracted data. Disagreements were resolved by discussion between the two review authors.

The following information was extracted from each included trial: the characteristics of the trial participants, the trial's inclusion and exclusion criteria, the type of intervention and outcome data. The chosen outcomes were defined as shown in Table 2.

To ascertain the validity of eligible studies, the two reviewers, working independently and with adequate reliability, measured the risk of bias; these data were assessed using the Newcastle-Ottawa Quality Assessment Scale 14 for cohort studies and the SIGN checklist 13. The critical evaluation of the included trials was expected to reveal a score ≥6, with a total of 9 being the highest possible score on the Newcastle-Ottawa Scale. The levels of evidence according to the Oxford Centre for Evidence-Based Medicine 15 were also obtained.

### Statistical analysis

The risk differences (RDs) for the complete primary resection, primary success, complication and recurrence rates after treatment of ampullary adenoma were the outcomes measured. In addition, data on absolute risk reduction (ARR) or increase (ARI) and the number needed to treat (NNT) or harm (NNH) were analyzed for the main outcomes. For all statistical calculations, we used a confidence interval (CI) of 95%.

The analysis was performed using the software Review Manager (RevMan) 5.3 16, obtained from the website of the Cochrane Informatics & Knowledge Management Department, by computing RDs for dichotomous variables using random- and fixed-effects models and generating the respective forest plots. Data on RDs and 95% CIs for each outcome were calculated using the Mantel-Haenszel test and inconsistency (heterogeneity) was quantified and reported using the Chi-squared (Chi^2^) test and the Higgins method and termed I^2^. The advantages of this last measure are that it does not inherently depend on the number of studies and that it is accompanied by an uncertainty interval.

As quantification of heterogeneity is only one component of a wider investigation of variability across studies and considering the clinical implications of the observed degree of inconsistency across studies, a cut-off value of 50% was assumed to be adequate for this meta-analysis 17. When the heterogeneity (I^2^) was greater than 50%, we compared the findings between the random- and fixed-effects models, and if no difference was found in the final results, true heterogeneity was presumed, i.e., significant publication bias was excluded.

Publication bias is concerned with what is likely to be published relative to what is available to be published. One problematic and much-discussed bias is the tendency of researchers and editors to handle the reporting of experimental results that are positive (i.e., showing a significant finding) differently from those results that are negative (i.e., supporting the null hypothesis) or inconclusive, leading to a misleading bias in the overall published literature 18.

## RESULTS

### Study identification and selection

**Figure 1-:**
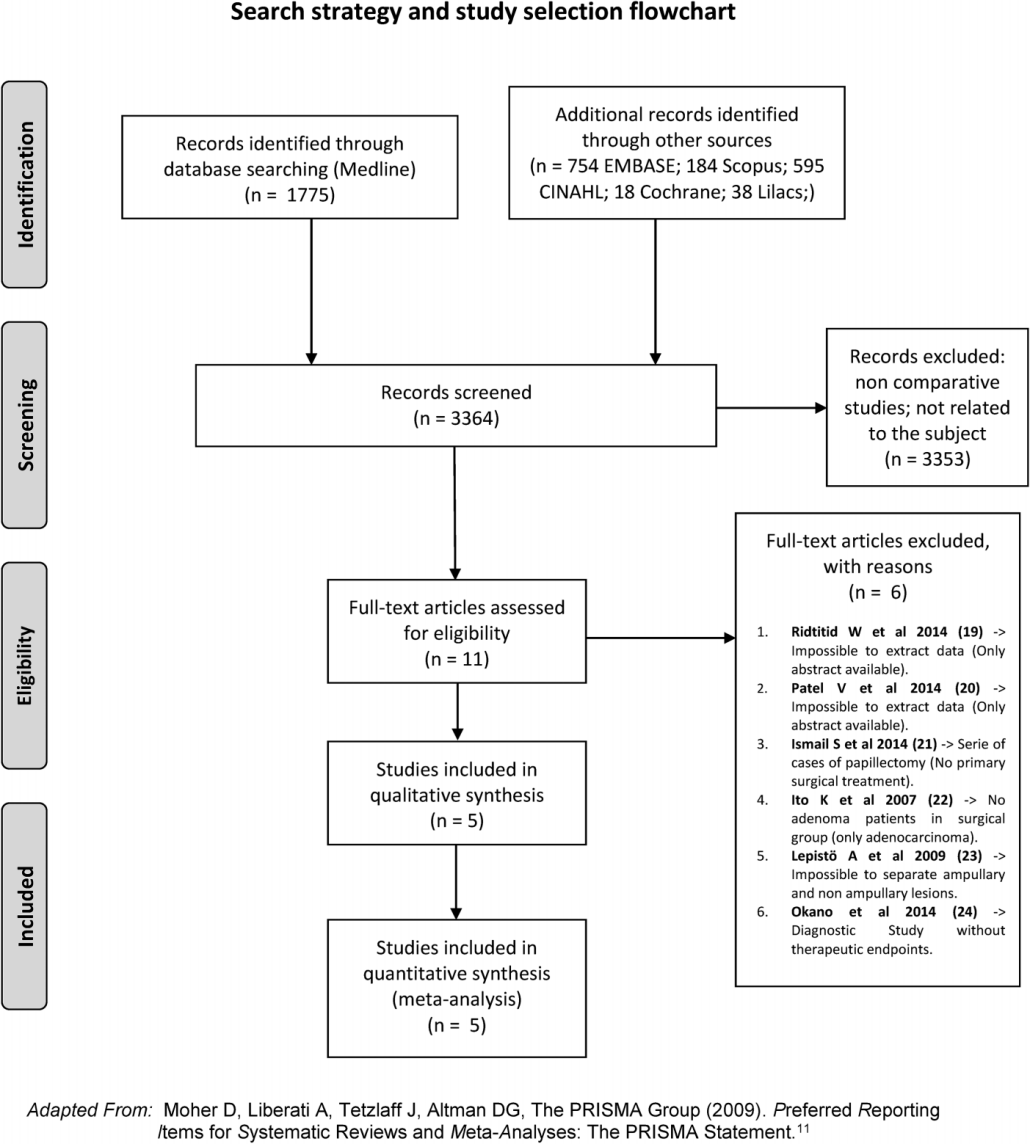
Search strategy and study selection flowchart.

Throughout the search strategy, three thousand, three hundred and sixty-four (3364) studies were screened and eligible articles were selected after the title and abstract were read. Three thousand, three hundred and fifty-three (3353) articles were excluded due to not being related to the subject or not being comparative studies. The full text of the eleven remaining studies was assessed for eligibility. Six articles were excluded: Ridtitid et al. 19 and Patel et al. 20 because only the abstract was available, so it was impossible to extract data; Ismail et al. 21 because a series of cases of papillectomy with no primary surgical treatment; Ito et al. 22 because adenoma patients were not included in the surgical group; Lepistö et al. 23 because of the impossibility of separating ampullary and non-ampullary lesions; and Okano et al. 24 because a diagnostic study without therapeutic endpoints was conducted. The remaining five studies were included in qualitative and quantitative syntheses. An adapted PRISMA flow diagram illustrates the study selection process (Figure 1).

### Study characteristics

#### Methods

The five articles selected for review were retrospective studies. All of them were published in English.

### Participants

The included records involved 466 patients. The main inclusion criterion was the presence of an ampullary adenoma that was managed with endoscopic or surgical treatment. All follow-up periods were considered.

### Intervention

The mainly endoscopic approach analyzed was endoscopic snare papillectomy (ESP). Complementary techniques such as hot biopsy (HB) and argon-plasma coagulation (APC) were also included.

### Comparison

The surgical approaches were PD and transduodenal resection (TDR), depending on the clinical condition of the patient. Both these approaches were together compared with the endoscopic approach.

### Outcomes

The assessed outcomes were the complete primary resection, primary success, recurrence and complication rates.

A summary of the characteristics of the included studies is shown in Table 3.

### Methodological quality and risk of bias

The risk of bias was assessed using a standard approach with defined criteria, as previously mentioned. Data from each selected study and the levels of evidence are shown in a tabular format (Table 4). All five included studies obtained a score of 8 according to the Newcastle-Ottawa Quality Assessment Scale (14) for cohort studies.

Other parameters were used to increase confidence in the strength of association between exposure and outcome by identifying aspects of good study design. According to the SIGN checklist (13), all studies were considered acceptable, i.e., having certain flaws and an associated risk of bias (the possibility of a change in the conclusions in light of further studies). The SIGN classification considers three category levels: 0 (low quality), + (acceptable) and ++ (high quality). All of the included studies were classified as having a level of evidence of 2B (Oxford Centre for Evidence-Based Medicine (15)).

### Analysis of outcomes

The data on effect estimates and CIs for each study are shown graphically. The numerical group-specific summary information, effect size, CI and percentage weight are also shown in the following tables (forest plots).

### Complete primary resection

**Figure 2-:**
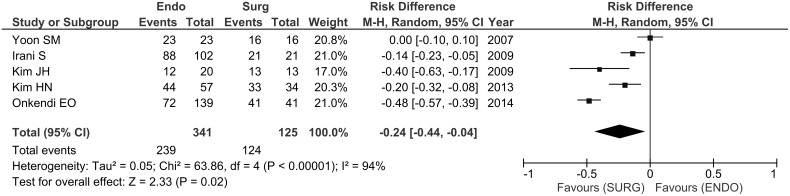
Complete primary resection data after endoscopic or surgical treatment of ampullary adenomas.

All five studies were available for the complete primary resection analysis. As shown in Figure 2, high heterogeneity was found (Chi^2^=63.83 and I^2^=94%), but no change in the effect was found in the comparison between the random- and fixed-effects models, indicating true heterogeneity between the studies. Data analysis showed a difference that favored surgical treatment (RD=-0.24, 95% CI=-0.44 to -0.04). The NNH calculated was 5, indicating that for every five patients who are endoscopically treated, one would be harmed in not undergoing complete resection (and would have benefited from surgical treatment with complete resection).

### Primary success

**Figure 3-:**
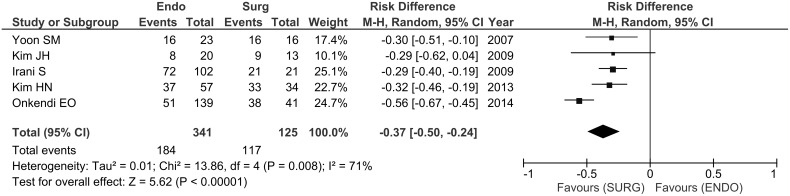
Primary success data after endoscopic or surgical treatment of ampullary adenomas.

Primary success data were identified in all five studies. The heterogeneity detected was higher than desirable (Chi^2^=13.86 and I^2^=71%) within this comparison (Figure 3), but the comparison of the effects models did not show a difference, implying true heterogeneity. Analysis of the 466 patients showed that the surgical approach was more successful than endoscopic treatment in terms of primary success (RD=-0.37, 95% CI=-0.50 to -0.24).

The pooled NNH was 3, showing that for every three patients who are endoscopically treated, one would not be cured by the first procedure and would need complementary treatment (and would have benefited from surgical treatment, with no need for additional resection).

### Recurrence

**Figure 4-:**
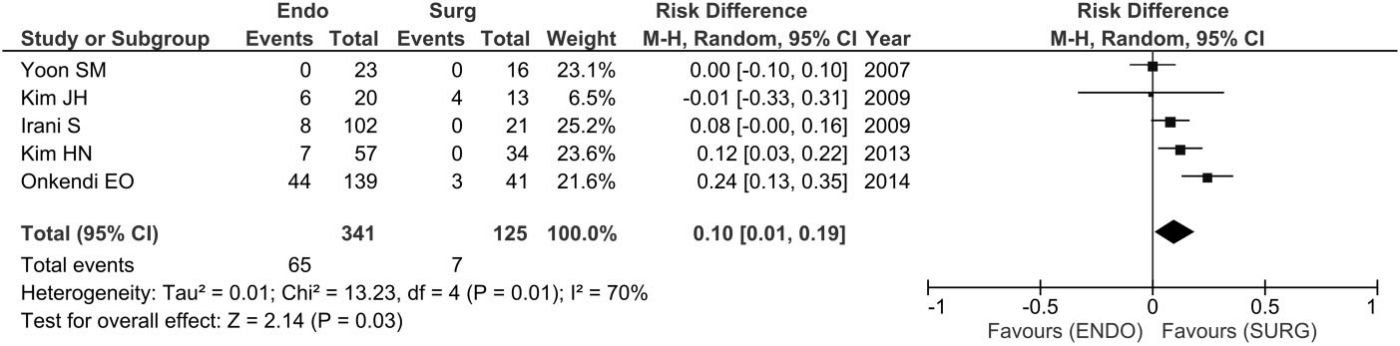
Recurrence data after endoscopic or surgical treatment of ampullary adenomas.

All five studies had recurrence data available for analysis. Although high heterogeneity was detected (Chi^2^=13.23 and I^2^=70%), as shown in Figure 4, it was considered to be true heterogeneity, as no difference in the result was found with the fixed-effects model. The data analysis showed that in the 466 patients, surgical treatment led to less recurrence in comparison with endoscopic treatment for ampullary adenomas (RD=0.10, 95% CI=0.01 to 0.19). The NNH calculated was 10, meaning that for every 10 patients who is treated with endoscopy, one would experience recurrence of the lesion in the follow-up period (this would not happen if surgical treatment was used).

### Complications

**Figure 5-:**
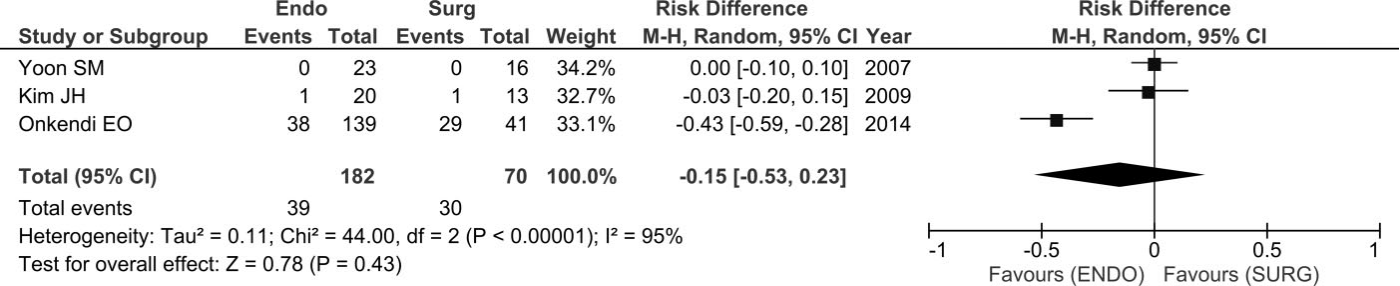
Complication data after endoscopic or surgical treatment of ampullary adenomas (random-effects model).

**Figure 6-:**
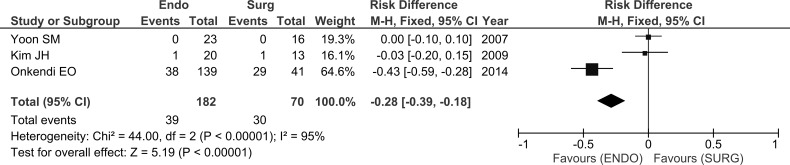
Complication data after endoscopic or surgical treatment of ampullary adenomas (fixed-effects model).

Complication data were identified for 254 patients (three studies). There was high heterogeneity in the sensitivity analysis (Chi^2^=44.00 and I^2^=95%). Random-effects model analyses showed no difference between surgical and endoscopic treatment (RD=-0.15, 95% CI=-0.53 to 0.23) (Figure 5), differing from what was found with the fixed-effects model, which showed fewer complications with the endoscopic approach (RD=-0.28, 95% CI=-0.39 to -0.18) (Figure 6). This finding, along with the high heterogeneity, suggests that publication bias may have been present.

## DISCUSSION

Ampullary adenomas are well known as premalignant lesions. An adenoma-to-carcinoma sequence similar to that in colonic cancer is well described (30) and because of that, resection is mandatory.

Endoscopic treatment for ampullary adenomas is well established by more than 30 years of experience all over the world, with the first description in 1983 by Suzuki et al. (31). ESP is a minimally invasive procedure with high success and low recurrence rates, although it has a significant risk of complications.

The advantage of the surgical approach via PD is that it is a ‘gold standard' procedure, with a historic legacy that extends from the late 19th century, and specifically 1889, when Codivilla reported the first PD. Over the following years, this approach's ominous prohibitive mortality was reduced and currently, its mortality rate is less than 2% in reference centers (32). Nevertheless, PD continues to be a very challenging and risky procedure and certain patients do not have adequate clinical conditions for this intervention. For example, in a study by Yoon et al., several patients were not managed surgically because of poor preoperative conditions, including advanced age and significant comorbidities (29).

The critical point in the management of these patients is the indication for endoscopic treatment. Although it is well understood that EP should be performed only when the adenoma is confined to the ampullary region, the specific indication criteria are not yet fully established (10,33). Summarizing all of the criteria utilized in the five studies included in the present review, we have derived the following contraindications for endoscopic treatment: endoscopic impression of malignancy (friability, ulceration, lateral spread, obvious duodenal infiltration), size >4 to 4.5 cm and intraductal extension of more than 1 cm on endoscopic retrograde cholangiopancreatography (ERCP) or EUS.

Another very important point in the management of these lesions is the reliability of the preoperative evaluation in terms of distinguishing between benign and malignant ampullary tumors. Certain authors have suggested that malignancy might be missed in up to 30% of tumors in the major duodenal papilla when forceps biopsy specimens are obtained 34. As the coexistence of carcinoma within adenoma cannot be completely excluded by pre-procedural biopsy, the use of ESP as a diagnostic tool (a ‘total biopsy') should be considered and would be useful as a screening method for identifying those patients who will need surgical treatment.

As shown in Table 3, the follow-up period was satisfactory for both interventions in all selected studies, with means of 32.9 months for the endoscopic approach and 48.2 months for the surgical approach. It is well established that a follow-up period of 6 months would be sufficient to define the absence of recurrence after treatment of ampullary adenomas 33,35.

The data analysis for primary success, with 466 patients, showed a significant difference that favored surgical treatment (RD=-0.37, 95% CI=-0.50 to -0.24 and *p*<0.0001). This result, with an NNH of 3, shows that for every three patients who are treated with endoscopy, one would be harmed, without achievement of a cure, if this is the first procedure (whereas the patient would benefit if treated surgically). The primary failings found in the follow-up period were when the patient experienced recurrence or residual disease. Residual disease was measured indirectly based on the complete primary resection rate. Data analysis for this outcome for the 466 patients in our review showed a significant difference that favored surgical treatment (RD=-0.24, 95% CI=-0.44 to -0.04 and *p*=0.02). This finding was expected, as the majority of the surgical procedures performed were PD, which extracts the entire duodenum. The recurrence data, with all 466 patients included, also showed better results for the surgical approach (RD=0.10, 95% CI=0.01 to 0.19 and *p*=0.03). Although surgical treatment showed less recurrence and residual disease, it is also important to highlight that when found after ESP, both of these findings are usually benign and most of them can be treated endoscopically 27,29,33,35.

As previously noted, ESP is considered as a procedure with a high risk of complications. In our analysis, only three studies provided complication data for both the surgical and the endoscopic approaches (Yoon et al., 2007; Kim et al., 2009; and Onkendi et al., 2014), with no significant difference between the two groups when using the random-effects model. However, using the fixed-effects model, the results showed a benefit for endoscopic treatment (RD=-0.28, 95% CI=-0.39 to -0.18 and *p*<0.00001). This finding, along with the high heterogeneity, did not permit the exclusion of publication bias in the studies by Yoon et al. and Kim et al., which showed a very low complication rate for both the endoscopic and the surgical approaches. As the only cases of complications reported in these two studies were related to mortality, it is reasonable to assume that minor complication data were not available or were even omitted. The other two studies (Kim HN et al., 2013 and Irani et al., 2009), which did not show complication data for the surgical approach, reported ESP complication rates of 28% and 20.5%, respectively, which are compatible with other findings in the literature, with complication rates for the endoscopic approach varying from 8 to 35% 33,36.

Undoubtedly, EP can be an effective primary therapy for ampullary adenoma and it is currently already an interesting option in many cases of ampullary adenoma. However, surgical treatment is very well established and there are no prospects of major changes in the coming years. In contrast, future evolution in the endoscopic area are a reality, and new technologies and standardization of techniques for endoscopic removal of ampullary adenoma may ensure more complete removal and minimal complications related to the endoscopic procedure, expanding the indications for EP.

As no guidelines are fully accepted regarding the treatment of ampullary adenomas, the management of this condition relies on the decision of the attending physician or medical staff and depends on the complex interaction of different factors, such as the patient's clinical condition and age, tumor characteristics, physician expertise (surgeons, pathologists and endoscopists) and hospital infrastructure. Regardless of the chosen technique, the patient should be treated in reference centers by trained and experienced professionals to ensure similar results as shown in this review.

The lack of studies comparing surgical and endoscopic treatment for ampullary adenoma is a fact. In the present study, the literature search represented a limitation, providing a low number of studies for analysis, with no randomized trials. The main reasons for this limitation are the absence of guidelines for the management of this disease and difficulty in defining the adequate indications for either surgery or EP.

The comparison between the findings with the random- and fixed-effects models showed a difference only in the complication outcome, meaning that publication bias could not be excluded. The presence of high heterogeneity and the lack of a difference in the comparison of the two effects models for the other outcomes indicate that true heterogeneity existed between the selected studies. A certain degree of heterogeneity is inevitable in a medical meta-analysis, but the impact of between-study heterogeneity may undermine the quality and legitimacy of the results obtained.

Considering complete primary resection, primary success and recurrence outcomes when comparing the endoscopic approach with the surgical approach for the treatment of ampullary adenomas, the surgical approach achieves significantly better results. Regarding complication data, this systematic review concludes that rates are not significantly different.

## AUTHOR CONTRIBUTIONS

Mendonça EQ is the main author. Bernardo WM is the peer reviewer. Moura EG is the scientific adviser. Chaves DM, Kondo A and Tao Pu L provided assistance during the manuscript writing. Baracat FI was responsible for the statistical analysis.

## Figures and Tables

**Table 1:** Eligibility criteria.

Types of studies	Comparative studies (clinical trials and/or observational studies)
Types of participants	Patients who had been diagnosed with ampullary adenoma
Types of interventions	Trials comparing outcomes between two groups (endoscopic treatment and surgery). There were no restrictions regarding the different modalities of treatment in each group
Types of outcomes or outcome measures	The main outcome measures were complete resection, primary success, recurrence and complications related to the procedures

**Table 2:** Outcomes.

Complete primary resection	The first procedure was able to completely extract the neoplasia, with free margins
Primary success	The initial procedure was sufficient to achieve complete cure of the adenoma within the follow-up period
Recurrence	After primary complete resection, the patient appeared to undergo a relapse of the adenoma, which was detected during the follow-up
Complication	Complications related to the procedure were present

**Table 3:** Summary of the included studies.

Study	Study design	Number of patients	Endoscopic approach (EA)	Surgical approach (SA)	Follow-up (median) EA / SA	Outcomes
Onkendi EO et al., 2014 (25)	RC	180	ESP; APC	PD; TDR	48.4 mo (*SDNA*)	CPR; PS; R; C;
Kim HN et al., 2013 (26)	RC	91	ESP; HB	*DNA*	26.6 mo / 26.6 mo	CPR; PS; R; C;
Irani S et al., 2009 (27)	RC	123	ESP; APC	PD	40 mo / *DNA*	PS; C;
Kim JH et al., 2009 (28)	RC	33	ESP	PD; TDR	20 mo / 60.6 mo	CPR; PS; R;
Yoon SM et al., 2007 (29)	RC	39	ESP; APC	PD	29.6 mo / 57.1 mo	CPR; PS; R; C;

RC: Retrospective Cohort; CPR: Complete Primary Resection; PS: Primary Success; R: Recurrence; C: Complications; ESP: Endoscopic Snare Papillectomy; HB: Hot Biopsy; APC: Argon-Plasma Coagulation; PD: Pancreaticoduodenectomy; TDR: Transduodenal Resection; mo: Months; DNA: Data Not Available; SDNA: Separate Data Not Available.

**Table 4:** Quality measures of the analyzed studies: Newcastle-Ottawa Scale and bias measures.

Study	Representativeness of exposed cohort and selection of non-exposed cohort (max. 2 points)	Ascertainment of exposure (max. 1 point)	Demonstration that outcome of interest was not present at start of study (max. 1 point)	Comparability of cohorts on basis of design or analysis (max. 2 points)	Assessment of outcome (max. 1 point)	Length and adequacy of follow-up (max. 2 points)	Score and level of evidence Ф
Onkendi EO et al., 2014 (25)	2	1	1	1	1	2	8 – 2B - acceptable (+)
Kim HN et al., 2013 (26)	2	1	1	1	1	2	8 – 2B - acceptable (+)
Irani S et al., 2009 (27)	2	1	1	1	1	2	8 – 2B - acceptable (+)
Kim JH et al., 2009 (28)	2	1	1	1	1	2	8 – 2B - acceptable (+)
Yoon SM et al., 2007 (29)	2	1	1	1	1	2	8 – 2B - acceptable (+)

Ф Oxford Centre for Evidence-Based Medicine and Scottish Intercollegiate Guidelines Network ratings.
